# Supplementary material for: The dmsEFABGH operon encodes an essential and modular electron transfer pathway for extracellular iodate reduction by Shewanella oneidensis MR-1
Source: Microbiol Spectr. 2024 Jun 25;12(8):e00512-24. doi: 10.1128/spectrum.00512-24 (PMC11302344; doi:10.1128/spectrum.00512-24)
Supplement: Supplemental table and figures — Tables S1; Fig. S1-S7. [file spectrum.00512-24-s0001.docx]

**Supplementary Figures**

**
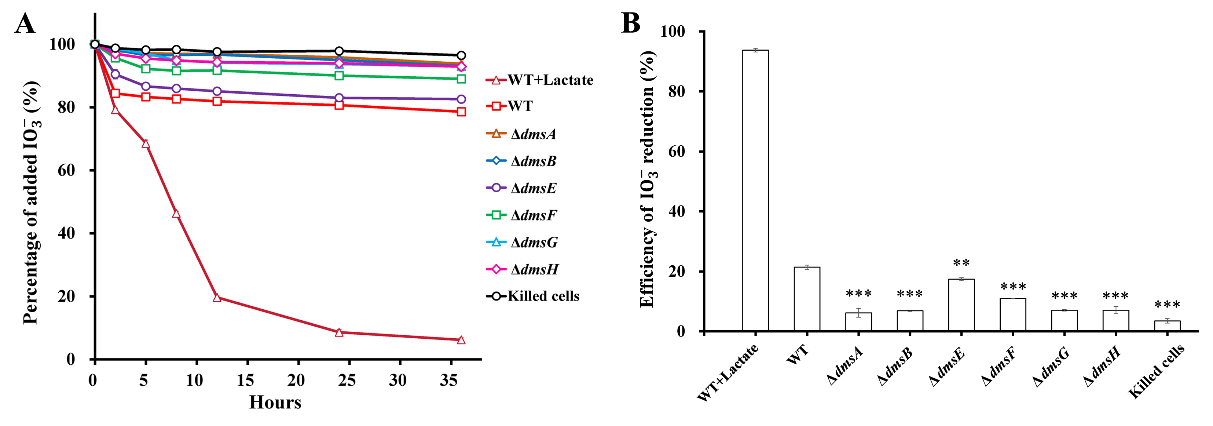
**

**Figure S1** $\mathbf{IO}_{\mathbf{3}}^{\mathbf{-}}$ **reduction by *S. oneidensis* MR-1 strains without electron donors.** (A) Percentage of added $\mathrm{IO}_{3}^{-}$ over 36 hours of reduction and (B) Efficiency of $\mathrm{IO}_{3}^{-}$ reduction at 36-hour. WT, *S. oneidensis* MR-1. One hundred percent of added $\mathrm{IO}_{3}^{-}$ was equal to 250 μM. The values reported are the means and standard deviations of triplicate experiments. For points without error bar, the error was smaller than the symbol. Asterisks, significance levels of difference between the mutants and WT, 0.01 < *P* < 0.05 (*), 0.001 < *P* < 0.01 (**), *P* < 0.001 (***).

**
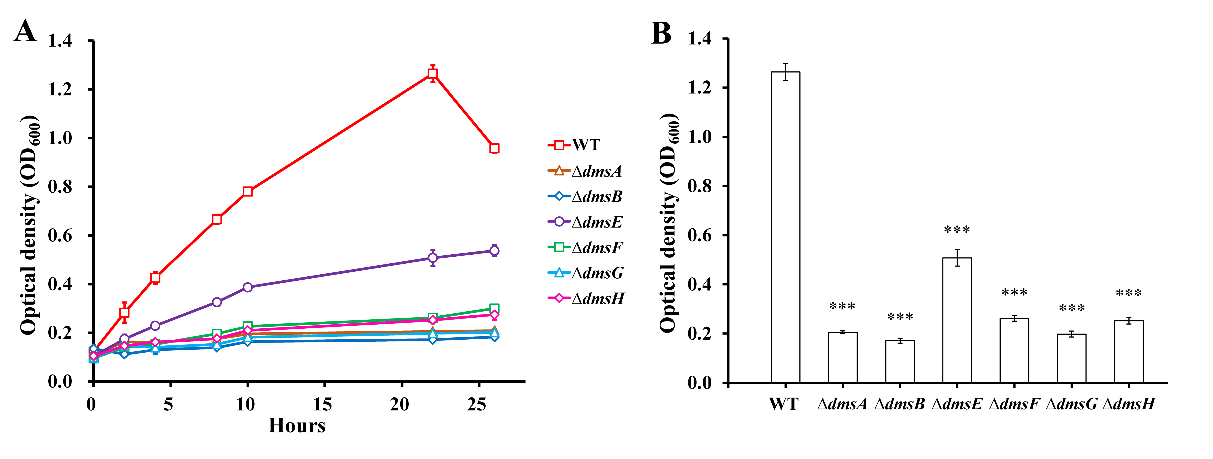
**

**Figure S2 Growth of the wild type and *dmsEFABGH* mutants with DMSO as the sole terminal electron acceptor.** (A) Growth over 26 hours. (B) Optical density at 22-hour. WT, S. oneidensis MR-1. The values reported are the means and standard deviations of triplicate experiments. For points without error bar, the error was smaller than the symbol. Asterisks, significance levels of difference between the mutants and WT, 0.01 < *P* < 0.05 (*), 0.001 < *P* < 0.01 (**), *P* < 0.001 (***).

**
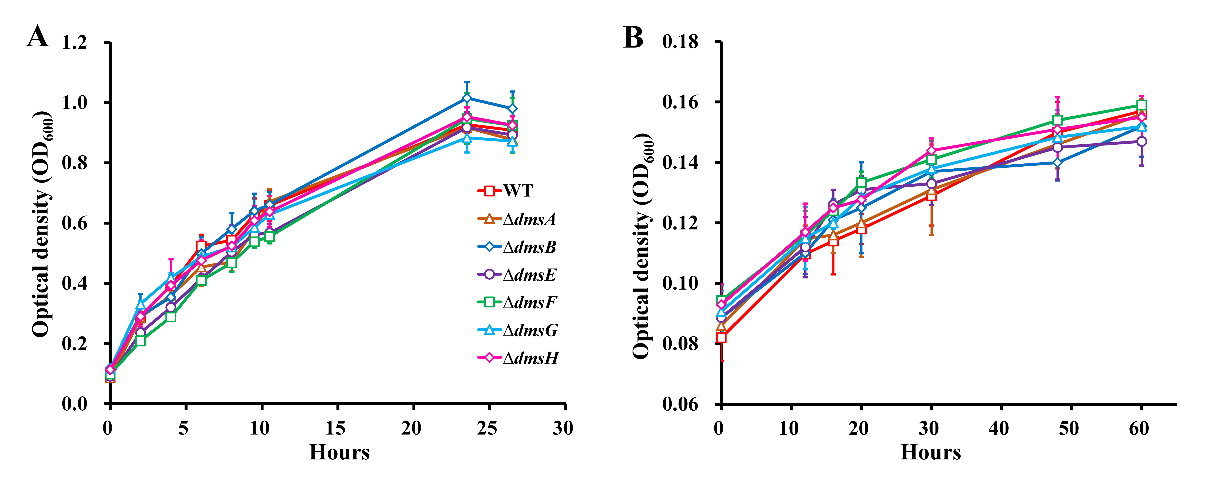
**

**Figure S3 Growth of the wild type and *dmsEFABGH* mutants with (A) O_2_ or (B) fumarate as the sole terminal electron acceptor.** WT, *S. oneidensis* MR-1. The values reported are the means and standard deviations of triplicate experiments. For points without error bar, the error was smaller than the symbol.

**
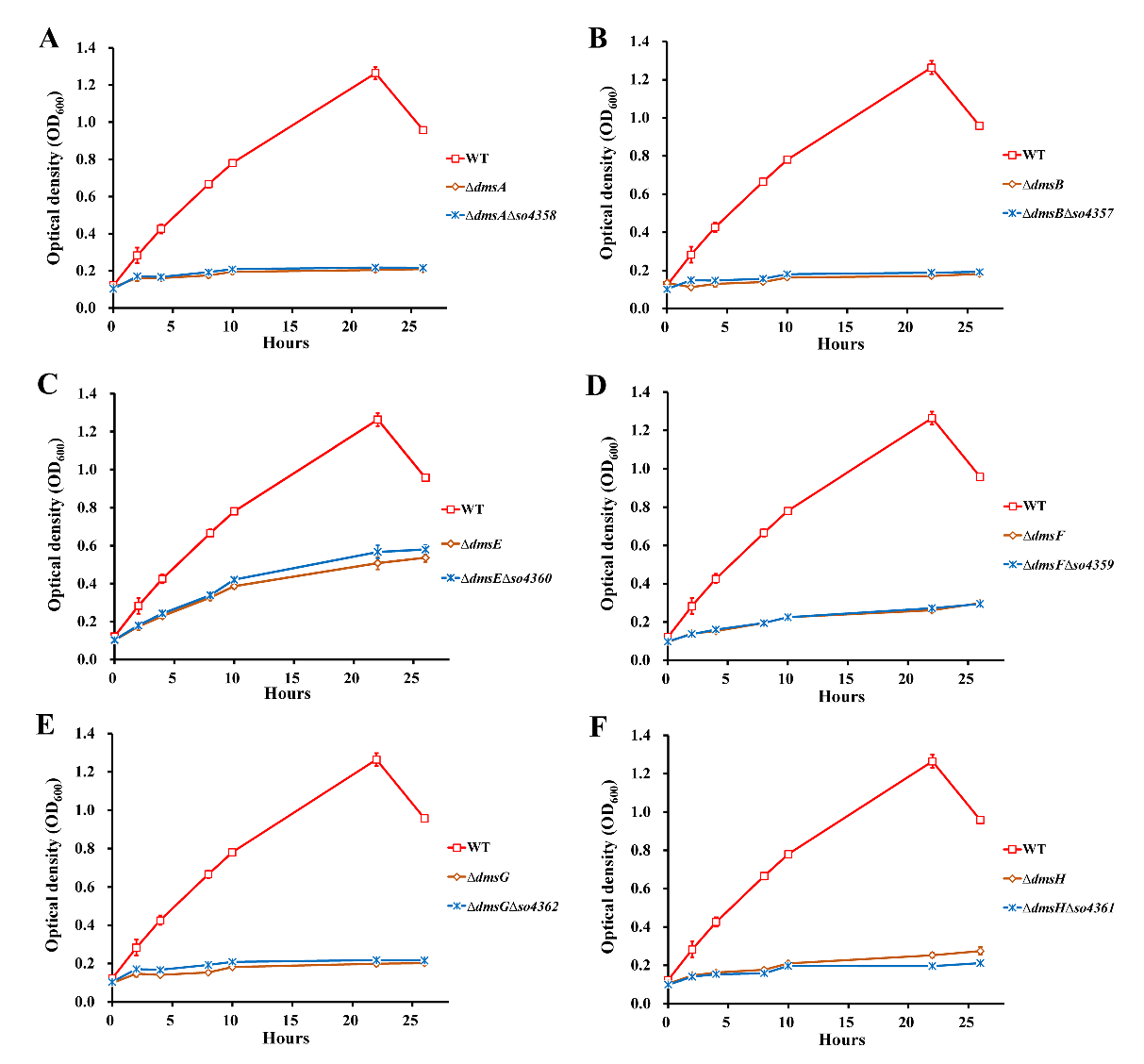
**

**Figure S4 Growth of the wild type, *dmsEFABGH* mutants and double-gene mutants over 26 hours with DMSO as the sole terminal electron acceptor.** WT, S. oneidensis MR-1. The values reported are the means and standard deviations of triplicate experiments. For points without error bar, the error was smaller than the symbol.

**
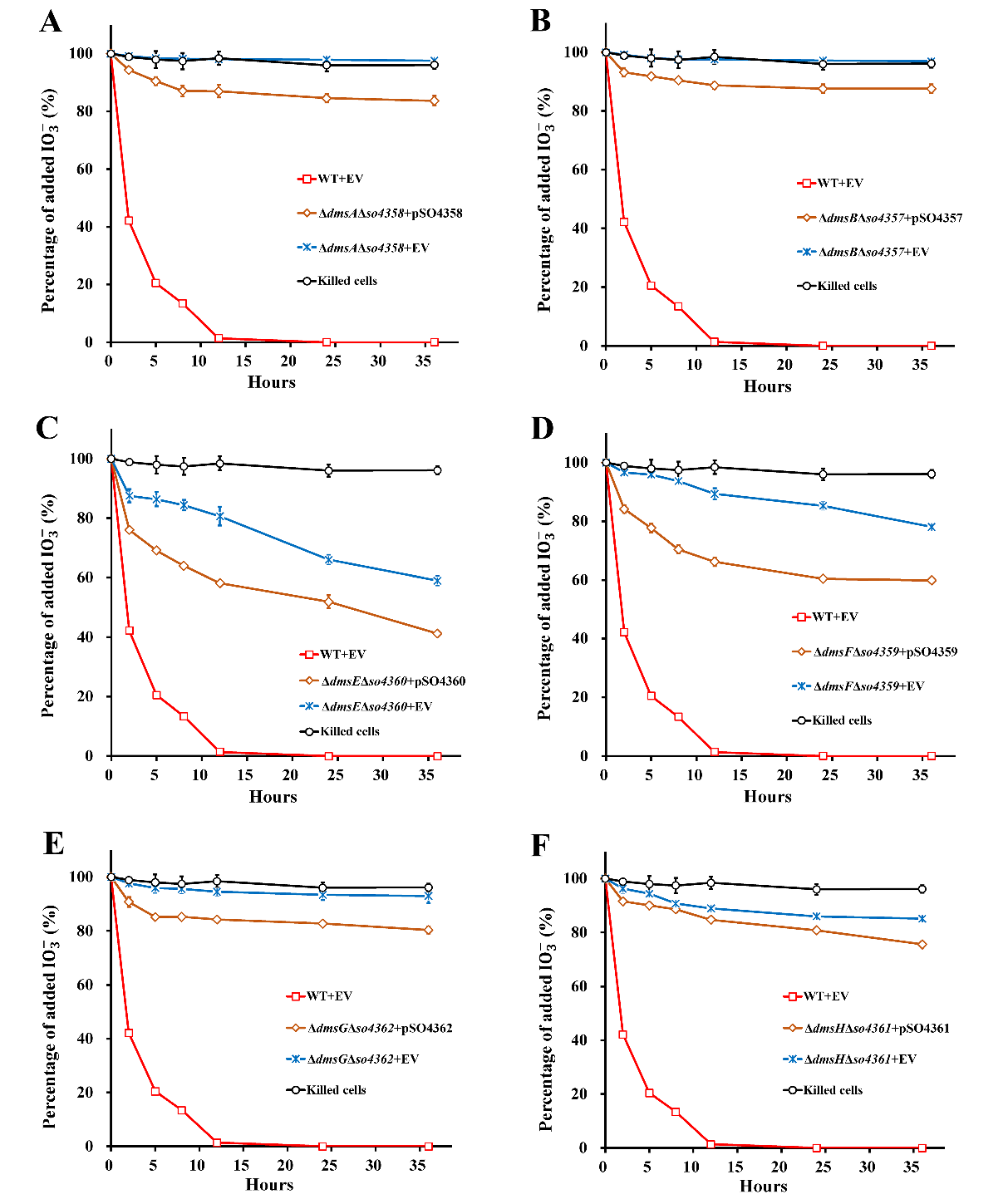
**

**Figure S5 Percentage of added** $\mathbf{IO}_{\mathbf{3}}^{\mathbf{-}}$ **over 36 hours of reduction by *dmsEFABGH* deletion mutants complemented with vectors containing respective *dms* genes or empty vector.** Lactate was used as the sole electron donor. WT, *S. oneidensis* MR-1; EV, empty vector pBBR1MCS-2. One hundred percent of added $\mathrm{IO}_{3}^{-}$ was equal to 250 μM. The values reported are the means and standard deviations of triplicate experiments. For points without error bar, the error was smaller than the symbol.

**
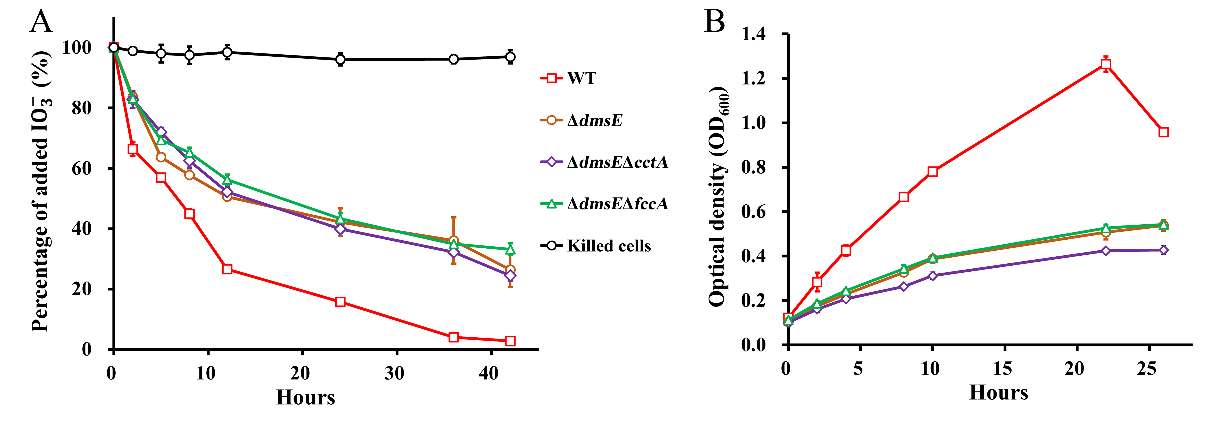
**

**Figure S6** $\mathbf{IO}_{\mathbf{3}}^{\mathbf{-}}$ **reduction by the wild type, Δ*dmsE*, Δ*dmsE*Δ*cctA*, and Δ*dmsE*Δ*fccA* and their growth.** (A) Percentage of added $\mathrm{IO}_{3}^{-}$ over 42 hours of reduction with lactate as the sole electron donor. (B) Growth over 26 hours with DMSO as the sole terminal electron acceptor. WT, S. oneidensis MR-1. One hundred percent of added $\mathrm{IO}_{3}^{-}$ was equal to 250 μM. The values reported are the means and standard deviations of triplicate experiments. For points without error bar, the error was smaller than the symbol.


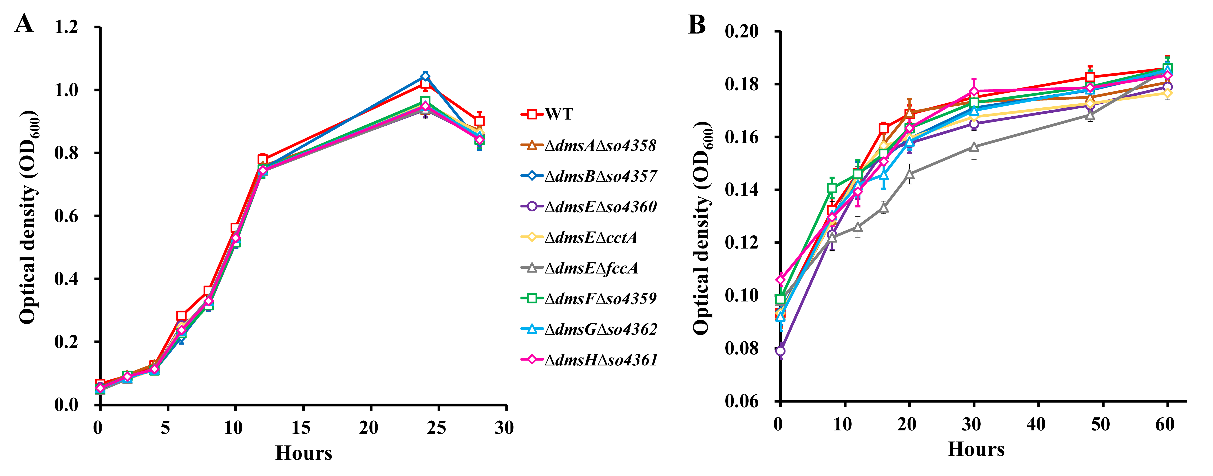


**Figure S7 Growth of the wild type and double-gene mutants with (A) O_2_ or (B) fumarate as the sole terminal electron acceptor.** WT, wild type of *S. oneidensis* MR-1. The values reported are the means and standard deviations of triplicate experiments. For points without error bar, the error was smaller than the symbol.

Table S1 Primers used in this study

| **Primers** | **Sequence (5’—3’)** | **Length of target sequences (bp)** | **Purpose** |
| --- | --- | --- | --- |
| dmsA5O | CGTTGCATGCTGCACCTCTATGCCTCAG | 668 | Construction of Δ*dmsA* |
| dmsA5I | GACTGGCTTAGGTCGTCTCTATGCCATCACGACTAAATAC |  |  |
| dmsA3I | AGAGACGACCTAAGCCAGTCTAAGAGGTGGCGATAAAGT | 763 |  |
| dmsA3O | AGTTGCATGCAAGAATCAACGCAAATAGG |  |  |
| dmsAFO | GCAGTTACCTTGAGCGAGAA | 4216 (WT), 2182 (Mutant) |  |
| dmsARO | CTGGCTTGCTAACTCATCTCG |  |  |
| dmsB5O | CTTGCCTGCAGGACAAATAGCGCGAATACG | 698 | Construction of Δ*dmsB* |
| dmsB5I | TTACACTTCTGCAGGGTTTATATTGTGTTTGTTGAGTCAT |  |  |
| dmsB3I | ATGACTCAACAAACACAATATAAACCCTGCAGAAGTGTAA | 609 |  |
| dmsB3O | ACTTCCTAGGGAGGTATCACATCAAACTTTGTA |  |  |
| dmsBFO | GCCGTTAAGCCTATGTGG | 2328 (WT), 1693 (Mutant) |  |
| dmsBRO | CACAAGCAGCCAAACTCA |  |  |
| dmsE5O | CTTGCCTGCAGGGATTTCACTGAAACAGGCGA | 613 | Construction of Δ*dmsE* |
| dmsE5I | TCAACGGGCAAAATTGCTGCTTAATTTTACGCCATCTCAT |  |  |
| dmsE3I | ATGAGATGGCGTAAAATTAAGCAGCAATTTTGCCCGTTGA | 533 |  |
| dmsE3O | ACTTCCTAGGAGCTCGCTTAACCAATTTTC |  |  |
| dmsEFO | ACAGTAGCCACCCGTTCA | 2505 (WT), 1588 (Mutant) |  |
| dmsERO | CATCGGGCAAGTAAGGTAA |  |  |
| dmsF5O | CTTGCCTGCAGGAATGTGAGGCATGTCATGG | 699 | Construction of Δ*dmsF* |
| dmsF5I | TCAAGGCAGCAGGTAGCTGAATATTTAGTTTAAAAGACAT |  |  |
| dmsF3I | ATGTCTTTTAAACTAAATATTCAGCTACCTGCTGCCTTGA | 720 |  |
| dmsF3O | ACTTCCTAGGCCCACTTTGACGCATTTC |  |  |
| dmsFFO | TCACGAAACGGGAGAACA | 3639 (WT), 1719 (Mutant) |  |
| dmsFRO | GGGTCCACCATAATCACTTC |  |  |
| dmsG5O | CTTGCCTGCAGGCGATAGCAGCAAATGTACTG | 664 | Construction of Δ*dmsG* |
| dmsG5I | TTAGCGATAGAGTTTTACAGGCTCGTATTGATTGTTAACAT |  |  |
| dmsG3I | ATGTTAACAATCAATACGAGCCTGTAAAACTCTATCGCTAA | 630 |  |
| dmsG3O | ACTTCCTAGGAAGGCCTGCCACGATGT |  |  |
| dmsGFO | TGTGGCTTTAGGTCAGGG | 2371 (WT), 1745 (Mutant) |  |
| dmsGRO | ATCAAGCGTGACCGAAGA |  |  |
| dmsH5O | CTTGCCTGCAGGTTCTTCGTTTGTAAAAGGAA | 585 | Construction of Δ*dmsH* |
| dmsH5I | TTATCCACCCATGTTTCGGGACTGCTGTCACAAACGTCAT |  |  |
| dmsH3I | ATGACGTTTGTGACAGCAGTCCCGAAACATGGGTGGATAA | 657 |  |
| dmsH3O | ACTTCCTAGGAGTAAATCAATTGAAAGACTTTTC |  |  |
| dmsHFO | TTTGCGTTGATTCTTGTCC | 2281 (WT), 1853 (Mutant) |  |
| dmsHRO | AATAGCGACCGCACTCAA |  |  |
| 4357-5O | CGTTCCTGCAGGGCGTAACCTTGAATACTCCT | 630 | Construction of Δ*dmsB*Δ*SO4357* |
| 4357-5I | GACTGGCTTAGGTCGTCTCTCCTTACAGGCCACTTGAC |  |  |
| 4357-3I | AGAGACGACCTAAGCCAGTCGGGAGATTATTAACATCACAGA | 641 |  |
| 4357-3O | AGTTCCTAGGTTTAGGCTACCAAACCAGA |  |  |
| 4357-FO | TCGGGAATGATGGTGTATT | 2300 (WT), 1813 (Mutant) |  |
| 4357-RO | TCGCTGTAGGCCCAGTAG |  |  |
| 4358-5O | CGTTGCATGCAGCTCAGAATTTATGCCAATG | 530 | Construction of Δ*dmsA*Δ*SO4358* |
| 4358-5I | GACTGGCTTAGGTCGTCTCTAGAACCACAACCCACAACG |  |  |
| 4358-3I | AGAGACGACCTAAGCCAGTCTGCTAGTTAGTCGCAAAGC | 648 |  |
| 4358-3O | AGTTGCATGCTTGGGTGAGTAATAGAAGATGA |  |  |
| 4358-FO | TCAAGAGCGTGAAGAGGTT | 4084 (WT), 1667 (Mutant) |  |
| 4358-RO | TACTGAGTTAATATGGATAGCG |  |  |
| 4359-5O | CGTTCCTGCAGGCGCCATTGTTATGTCAGC | 577 | Construction of Δ*dmsF*Δ*SO4359* |
| 4359-5I | GACTGGCTTAGGTCGTCTCTAGCATTGTTCGCATCTTG |  |  |
| 4359-3I | AGAGACGACCTAAGCCAGTCATACTCGCTTAGGATTGACTG | 601 |  |
| 4359-3O | AGTTCCTAGGGAACCACAACCCACAACG |  |  |
| 4359-FO | AACAAATGGGCAGATGAC | 2640 (WT), 1606 (Mutant) |  |
| 4359-RO | GCTACGGGTAAATAAATGG |  |  |
| 4360-5O | CGTTCCTGCAGGACGACGATAGCGAGTATTA | 566 | Construction of Δ*dmsE*Δ*SO4360* |
| 4360-5I | GACTGGCTTAGGTCGTCTCTAGGTATCTGCACCACTCTTA |  |  |
| 4360-3I | AGAGACGACCTAAGCCAGTCCGCCATTGTTATGTCAGC | 577 |  |
| 4360-3O | AGTTCCTAGGAGCATTGTTCGCATCTTG |  |  |
| 4360-FO | TTTTATCAGGCTATTGCTG | 2055 (WT), 1463 (Mutant) |  |
| 4360-RO | ACTCATCCCTTTGTTTGTAT |  |  |
| 4361-5O | CGTTCCTGCAGGATCACAAGGCTGCTAAAT | 503 | Construction of Δ*dmsH*Δ*SO4361* |
| 4361-5I | GACTGGCTTAGGTCGTCTCTGCTAATACTCGCTATCGTC |  |  |
| 4361-3I | AGAGACGACCTAAGCCAGTCTTGCGTCGGATGTATTTG | 646 |  |
| 4361-3O | AGTTCCTAGGGACTTTCTGCTTGGTGGC |  |  |
| 4361-FO | ATATGCAGGTGCTGGGTC | 1945 (WT), 1700 (Mutant) |  |
| 4361-RO | CAATGGCGCTCTGGTCTT |  |  |
| 4362-5O | CGTTCCTGCAGGGCGACACCCTTGAGTTCG | 611 | Construction of Δ*dmsG*Δ*SO4362* |
| 4362-5I | GACTGGCTTAGGTCGTCTCTAAGCATGGACCCAGCACC |  |  |
| 4362-3I | AGAGACGACCTAAGCCAGTCGCTATTGCTGGGCTTTCC | 509 |  |
| 4362-3O | AGTTCCTAGGTACATCCGACGCAACTAA |  |  |
| 4362-FO | GATTTGCGTGCGGTCTTC | 2020 (WT), 1515 (Mutant) |  |
| 4362-RO | GGCAGGTGAGGCAGGTAT |  |  |
| fccA-5O | CTTGCCTGCAGGGGCGACTAAGTCGACATTGT | 564 | Construction of  Δ*dmsE*Δ*fccA* |
| fccA-5I | CGTTAGCTGCAGACATAGGAATCTGATATCGTCACCTACGG |  |  |
| fccA-3I | TTCCTATGTCTGCAGCTAACGCAGAGATCAGCATGGCTAGT | 559 |  |
| fccA-3O | AAGTCCTAGGGCCCAGACATAGACTTAACC |  |  |
| fccA-FO | CGAGCAGCACCTTAATGGTT | 3246 (WT), 1587 (Mutant) |  |
| fccA-RO | CTGAATGAGCCAACGCCAAT |  |  |
| cctA-5O | CTTGCCTGCAGGCGAGTTGCAGGCTTATCAAG | 568 | Construction of  Δ*dmsE*Δ*cctA* |
| cctA-5I | CGTTAGCTGCAGACATAGGAAATGACGATGGCCGTACTTCT |  |  |
| cctA-3I | TTCCTATGTCTGCAGCTAACGACAGCGCTAACGCTGCAAGA | 604 |  |
| cctA-3O | AAGTCCTAGGCACGCATTCGTGAATATCGC |  |  |
| cctA-FO | GGCATATCTGAACTCTCAGG | 1698 (WT), 1466 (Mutant) |  |
| cctA-RO | CGTATCGAGCAACGATGAAG |  |  |
| CdmsAF | CTAGGAATTC*TAAGAAGGAGATATACATCCC*ATGGAACGCAGAAGTTTTCTA | 2511 | Construction of pDmsA |
| CdmsAR | CATGTCTAGATTATGCCTTGACGATCTGCAC |  |  |
| CdmsBF | CCGGAATTC*GAAAGAGGAGAAA*TACTAGATGACTCAACAAACACAATAT | 675 | Construction of pDmsB |
| CdmsBR | TGCTCTAGATTACACTTCTGCAGGGTTTAA |  |  |
| CdmsEF | CCGGAATTC*GAAAGAGGAGAAA*TACTAGATGAGATGGCGTAAAATTAAA | 957 | Construction of pDmsE |
| CdmsER | TGCTCTAGATCAACGGGCAAAATTGCTGCC |  |  |
| CdmsFF | CCGGAATTC*GAAAGAGGAGAAA*TACTAGATGTCTTTTAAACTAAATATC | 1989 | Construction of pDmsF |
| CdmsFR | TGCTCTAGATCAAGGCAGCAGGTAGCTGA |  |  |
| CdmsGF | CCGGAATTC*GAAAGAGGAGAAA*TACTAGATGTTAACAATCAATACGAGT | 666 | Construction of pDmsG |
| CdmsGR | TGCTCTAGATTAGCGATAGAGTTTTACAGG |  |  |
| CdmsHF | CCGGAATTC*GAAAGAGGAGAAA*TACTAGATGACGTTTGTGACAGCAGTG | 468 | Construction of pDmsH |
| CdmsHR | TGCTCTAGATTATCCACCCATGTTTCG |  |  |
| C4357F | CCGGAATTC*GAAAGAGGAGAAA*TACTAGATGCAAGAACCAACCCAATAC | 618 | Construction of pSO4357 |
| C4357R | TGCTCTAGATTACACTTCTGTGATGTTAAT |  |  |
| C4358F | CCGGAATTC*GAAAGAGGAGAAA*TACTAGATGAAAAGACGTGCATTTTTGA | 2589 | Construction of pSO4358 |
| C4358R | TGCTCTAGATTAAGCCTGAACAATTCTGATACG |  |  |
| C4359F | CCGCTCGAG*GAAAGAGGAGAAA*TACTAGATGAAGTTAAGTAAAACGACA | 1971 | Construction of pSO4359 |
| C4359R | TGCGGATCCTTAAAAGCTTTTCTTATATAA |  |  |
| C4360F | CCGCTCGAG*GAAAGAGGAGAAA*TACTAGATGAAAAAAATACTTTTATTT | 915 | Construction of pSO4360 |
| C4360R | TGCGAATTCTTACTTCAGTAACTTATTGCC |  |  |
| C4361F | CCGGAATTC*GAAAGAGGAGAAA*TACTAGATGAGTTTACTGTTTACAGTG | 468 | Construction of pSO4361 |
| C4361R | TGCTCTAGATTATTTTGAAAGCATATTTTTG |  |  |
| C4362F | CCGGAATTC*GAAAGAGGAGAAA*TACTAGATGAGCAAATCCTCATTTGAC | 678 | Construction of pSO4362 |
| C4362R | TGCTCTAGATCAAATTGACCTAATATTTTTTAGA |  |  |
| pBBR-LP | TTGGGTAACGCCAGGGTTTT | 297 | pBBR1MCS-2 detection and sequencing |
| pBBR-RP | TGTGTGGAATTGTGAGCGGA |  |  |
| sbavF | CCTGCAGGTTCTCACCTAGGAGCT | 24 | Construction of pDS3.2 |
| sbavR | CCTAGGTGAGAACCTGCAGGCATG |  |  |
| pds3F2 | TGGCGAAGTAATCGCAACA | 419 | Vector sequencing |
| pds3R2 | CCAGGGATGTAACGCACTGA |  |  |

Note: Underline, restriction sites; SphI, GCATGC; EcoRI, GAATTC; XbaI, TCTAGA; SbfI, CCTGCAGG; AvrII, CCTAGG; BamHI, GGATCC; XhoI, CTCGAG. Italic, ribosomal binding sites.
